# Supplementary figures and images for: Modeling viral shedding and symptom outcomes in oseltamivir-treated experimental influenza infection
Source: PLoS One. 2026 Feb 10;21(2):e0342676. doi: 10.1371/journal.pone.0342676 (PMC12890086; doi:10.1371/journal.pone.0342676)

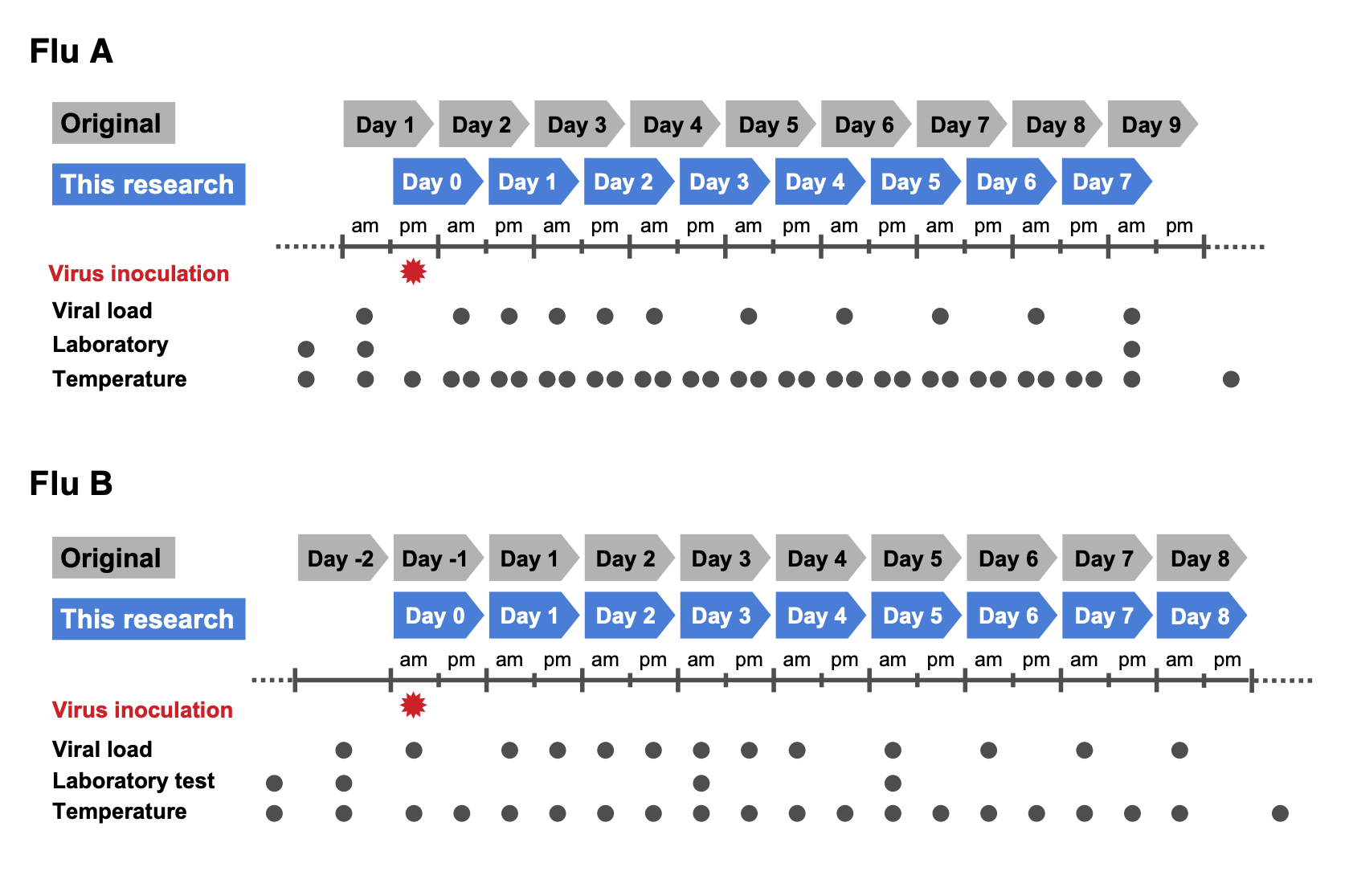

Supplement: S1 Fig — The schedule from which each datum point used in the analysis was measured in the clinical trials. Times were modified according to the time of inoculation and used for the data analysis. (TIF) [file pone.0342676.s001.tif]

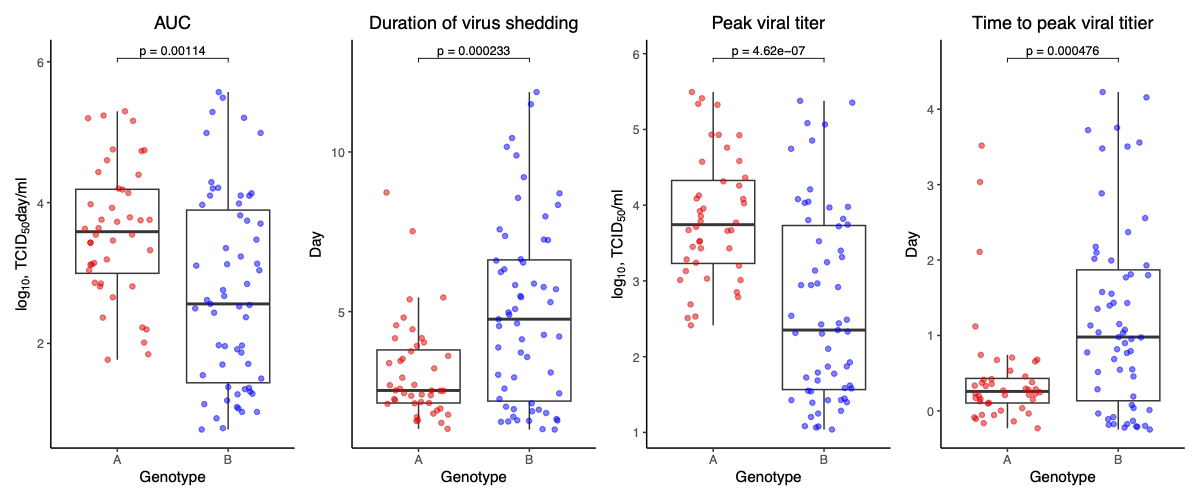

Supplement: S3 Fig — Comparison of estimated viral load-related outcomes, AUC, duration of virus shedding, peak viral titer, and time to peak viral titer between influenza A and B infection. The means were compared using a t test, and p-values were adjusted using a post hoc Bonferroni correction. (TIF) [file pone.0342676.s003.tif]

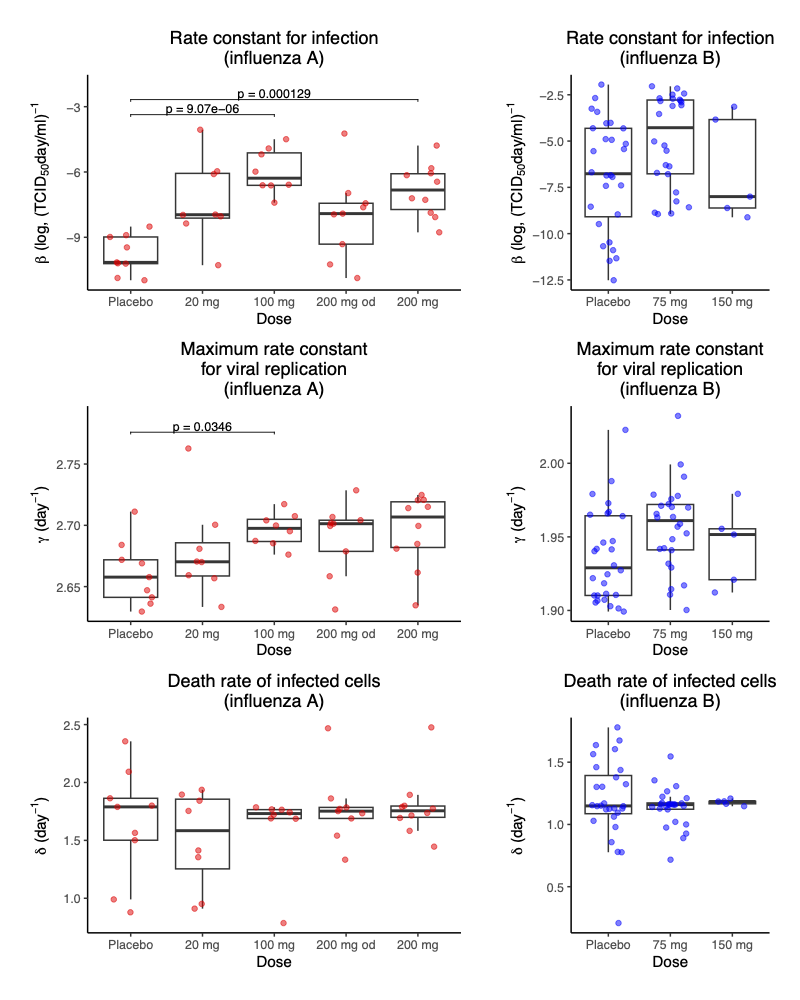

Supplement: S4 Fig — Comparison of the estimated model parameters between groups by dose of oseltamivir for influenza A (left) and influenza B (right) viruses. The doses without “od” were administered twice daily; the dose with “od” was administered once daily. We adjusted p-values according to the number of group combinations using a post hoc Bonferroni correction. (TIF) [file pone.0342676.s004.tif]
