# Supplementary material for: Modeling viral shedding and symptom outcomes in oseltamivir-treated experimental influenza infection
Source: PLoS One. 2026 Feb 10;21(2):e0342676. doi: 10.1371/journal.pone.0342676 (PMC12890086; doi:10.1371/journal.pone.0342676)
Supplement: S8 Table — Values of estimated population parameters, β, γ, δ, V(0), are listed in S8_Table. (PDF) [file pone.0342676.s012.pdf]

Name	Symbol	Unit	Population value	Variance of individual parameters	Covariate for genotype B (*p*-value by Wald test)
Rate constant for infection	β	(RNA copies/mL)$^{-1}$day^{-1}	5.55×10^{-4}	2.68	1.65 (1.37×10^{-2})
Maximum rate constant for viral replication	γ	Day^{-1}	14.7	9.55×10^{-2}	−0.739 (2.47×10^{-13})
Death rate of infected cells	δ	Day^{-1}	5.18	0.411	−0.595 (2.98×10^{-3})
Initial value of viral load	$V(0)$	$TCID_{50}$/mL	0.492	1.96	0.959 (0.659)
